# Immunomodulatory Potential of Non-Classical HLA-G in Infections including COVID-19 and Parasitic Diseases

**DOI:** 10.3390/biom12020257

**Published:** 2022-02-04

**Authors:** Sajad Rashidi, Carmen Vieira, Renu Tuteja, Reza Mansouri, Mohammad Ali-Hassanzadeh, Antonio Muro, Paul Nguewa, Raúl Manzano-Román

**Affiliations:** 1 Department of Parasitology and Mycology, School of Medicine, Shiraz University of Medical Sciences, Shiraz 7134845794, Iran; sajaderashidiii@gmail.com; 2 Infectious and Tropical Diseases Group (E-INTRO), Institute of Biomedical Research of Salamanca-Research Center for Tropical Diseases (IBSAL-CIETUS), Faculty of Pharmacy, University of Salamanca, 37008 Salamanca, Spain; carmelilla@usal.es (C.V.); ama@usal.es (A.M.); 3 Parasite Biology Group, ICGEB, Aruna Asaf Ali Marg, New Delhi 110067, India; renututeja@gmail.com; 4 Department of Immunology, Faculty of Medicine, Shahid Sadoughi University of Medical Sciences and Health Services, Yazd 8915173143, Iran; Rmansouri@ssu.ac.ir; 5 Department of Immunology, School of Medicine, Jiroft University of Medical Sciences, Jiroft 7861615765, Iran; m.hassanzadeh@jmu.ac.ir; 6 Department of Microbiology and Parasitology, ISTUN Institute of Tropical Health, IdiSNA (Navarra Institute for Health Research), University of Navarra, c/ Irunlarrea 1, 31008 Pamplona, Spain

**Keywords:** human leukocyte antigen-G, immune-regulation, co-infections, *Plasmodium falciparum*, COVID-19

## Abstract

Human Leukocyte Antigen-G (HLA-G), a polymorphic non-classical HLA (HLA-Ib) with immune-regulatory properties in cancers and infectious diseases, presents both membrane-bound and soluble (sHLA-G) isoforms. Polymorphism has implications in host responses to pathogen infections and in pathogenesis. Differential expression patterns of HLA-G/sHLA-G or its polymorphism seem to be related to different pathological conditions, potentially acting as a disease progression biomarker. Pathogen antigens might be involved in the regulation of both membrane-bound and sHLA-G levels and impact immune responses during co-infections. The upregulation of HLA-G in viral and bacterial infections induce tolerance to infection. Recently, sHLA-G was found useful to identify the prognosis of *Coronavirus* disease 2019 (COVID-19) among patients and it was observed that the high levels of sHLA-G are associated with worse prognosis. The use of pathogens, such as *Plasmodium falciparum*, as immune modulators for other infections could be extended for the modulation of membrane-bound HLA-G in COVID-19-infected tissues. Overall, such information might open new avenues concerning the effect of some pathogens such as parasites in decreasing the expression level of HLA-G to restrict pathogenesis in some infections or to influence the immune responses after vaccination among others.

## 1. Introduction

Major Histocompatibility Complex (MHC or the synonymous HLA, Human Leucocyte Antigens) has three regions designated as Class I, Class II, and Class III, and genes belonging to each of them encode the synthesis of molecules with different structure and function. The main function of class I gene products is the presentation of endogenous peptide antigens to CD8^+^ T cells. The HLA-I molecules are classified into classical HLA-Ia (HLA-A, B, and C) and non-classical HLA-Ib (HLA-E, F, and G). The classical molecules show high polymorphism and are characterized by unique patterns of transcription, protein structure, and immunological function. Non-classical MHC-I molecules exert functions in both the innate and adaptive immune system and, compared to classical MHC-I, appear to have mostly inhibitory effects on immune cells via interaction with inhibitory receptors [1].

HLA-G plays a major role in the down-regulation of the functions of the innate and adaptive immune system cells through interaction with multiple inhibitory receptors such as Leukocyte Immunoglobin-Like Receptors (LILRs) and in the presentation of non-peptidic antigens to non-classical T cells, leading to unique and fast immune responses upon infections [2]. The origin, structure, and functions of this molecule have been recently published. As of September 2020, the IMGT-HLA database includes 80 HLA-G alleles, encoding 21 complete and 4 truncated proteins (HLA-G1*01:05N, G*01:13N, G*01:21N and G*01:25N) [3,4]. Four membrane-bound (HLA-G1-4) and three soluble HLA-G isoforms (sHLA-G5-7) have been identified, each with different globular domains, mainly α1, α2, α3, and a beta-2-microglobulin (β2M) in HLA-G1 and HLA-G5 isoforms, the most important ones [5,6]. HLA-G1 isoform is found in soluble form due to proteases shedding. Their potential to dimerize plays important physiological roles for both soluble and membrane-bound HLA-Gs including higher affinity and avidity for their corresponding inhibitory receptors. Specially, the HLA-G1 homodimer orientation and the presence or absence of β2M-free isoforms appear essential to provide the required plasticity and/or flexibility of the complex states [7]. HLA-G does not seem to initiate immune responses as its classical homologues. In addition, a number of studies have highlighted the important role of this immune checkpoint molecule in the modulation of the immune system in immune-related diseases, tumors, and infections through the structure and dynamics of the different HLA-G isoforms [8,9,10,11].

**Figure 1:**
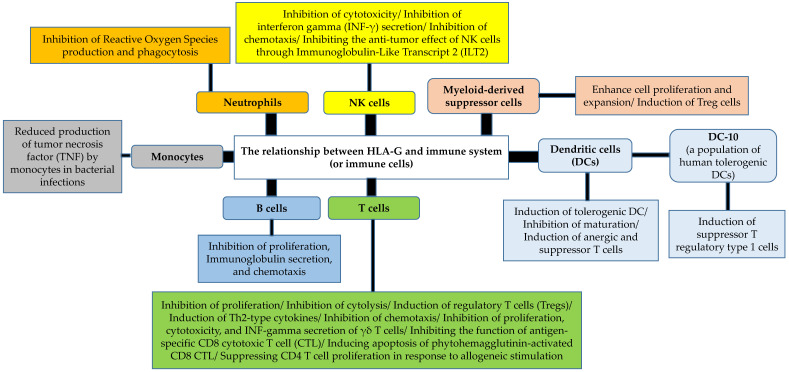
Various immune tolerogenic properties of HLA-G by modulating the functions of immune cells [15,19,20,21,22,23,24,25].

HLA-G is mainly expressed in the placenta, in embryonic tissues, in adult immune-privileged organs, and in cells of the hematopoietic lineage. HLA-G-positive (HLA-G^+^) CD4^+^ and CD8^+^ T lymphocytes, monocytes, and Natural Killer (NK) and dendritic cells are also detected in some patients with pathological disorders [12]. The molecule has a particular conformation that allows the presentation of a promiscuous repertoire of peptides similar to its functional homologue in mice, promoting a number of immunomodulatory effects as specific non-classical cytotoxic T cell responses rapidly responding to infections [13]. Accordingly, HLA-G^+^ cells control both the priming and the effector phases of the immune responses, therefore participating in peripheral immune tolerance, and these may be a target for strategies that have been aimed to develop immune checkpoint inhibitors (Figure 1) [12,14]. Thus, HLA-G^+^ immune cells are probably implicated in the complex mechanisms underlying the pathogenesis of various disorders including infections, transplants, cancers, and immune-mediated diseases [15,16,17,18].

## 2. Functions of HLA-G in Diseases

**Figure 2:**
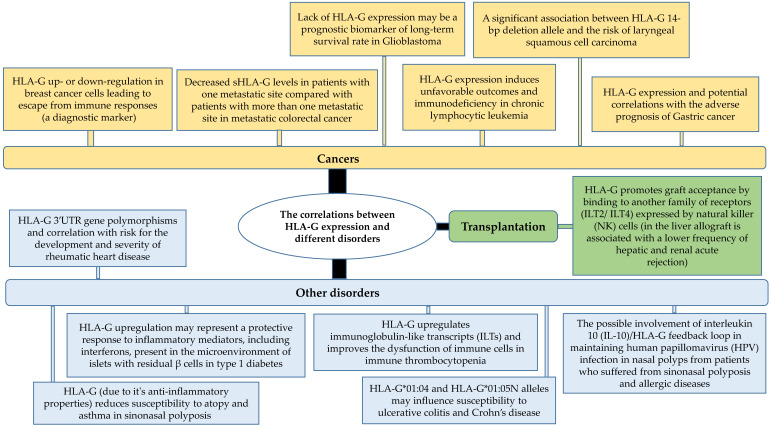
Correlation between HLA-G expression and different disorders.

It has been reported that the expression of HLA-G polymorphic variants could be associated with susceptibility to different diseases [26,27]. Figure 2 shows some correlations between HLA-G expression and several disorders especially cancers [1,12,19,21,24,26,28,29,30,31,32,33,34,35,36,37]. Furthermore, the expression of HLA-G has been described in several infectious diseases caused by bacterial, viral, or parasitic agents (Table 1) [16,38,39]. In viral infections, the overexpression of HLA-G can lead to the induction of a tolerogenic environment and to the inhibition of the immune response which makes it an immune escape mechanism. Moreover, differential expression patterns of sHLA-G or its polymorphisms seem to be related to different pathological conditions, potentially acting as a disease progression biomarker and a therapeutic target [40]. To this end, a number of different monoclonal antibodies (mAbs) able to recognize several HLA-G isoforms (mainly HLA-G1 and HLA-G5) must be assayed for diagnostic purposes due to its cross-reactivity, mainly with the β2M free classical HLA class I antigens; in particular, the 4H84 mAb and because different epitopes of HLA-G are detected by different monoclonal antibodies. One mAb, the MEM-G/9 is the option to simultaneously detect by an enzyme-linked immunosorbent assay (ELISA) captured β2M-related HLA-G1 shedding and HLA-G5 using a polyclonal anti-β2M antiserum [41,42,43].

Overall, HLA-G expression is upregulated in infectious diseases in response to changes in the cytokine microenvironment that is mainly related to increased levels of IL-10 and class I interferons. sHLA-G can be also expressed as a membrane-bound form on monocytes or different types of T cells (mainly CD4 and regulatory T cells (Tregs)) [12]. HLA-G is expressed in infected tissues and more frequently in peripheral blood, in the form of sHLA-G via alternative splicing or proteolytic cleavage, with a half-life between 6 and 24 h. Interestingly, the metalloproteases involved in the cleavage of the HLA-G membrane-bound molecule may act also as anti-inflammatory molecules [44].

HLA-G may have deleterious effects, promoting pathogen escape from immune system control (in cancers), or it may be beneficial (in septic shock), reflecting appropriate and effective feedback control of inflammatory processes [45,46]. HLA-G can be a single marker of infectious diseases when dealing with pathogens and/or to the immune response, or it may constitute a therapeutic target, once its function has been clarified in particular types of infections [38].

Investigations into sHLA-G, including purified sHLA-G1 protein and sHLA-G-linked extracellular vesicles, revealed the potential role of sHLA-G in mediating immune-modulatory activities in different diseases towards differential modifications in the phenotype of CD8 T cells [20,47] and suggested it as a potential diagnostic and prognostic biomarker even being implicated in the predisposition to infection [11,48,49]. Both membrane-bound and sHLA-G induce regulatory mechanisms, such as apoptosis of CD8^+^ T and NK cells, inhibition of B-cell proliferation, differentiation, and antibody secretion [50,51]. For instance, the up-regulation of HLA-G in tumors and the enhanced serum levels of soluble form have been identified in malignancies, probably leading to tumoral immune evasion and cancer progression due to their inhibitory actions on the immune system [52].

Severe Acute Respiratory Syndrome Coronavirus 2 (SARS-CoV-2) is currently a threat to human life worldwide. It is well known that different HLA alleles can affect antigen presentation and thus both the exposure and the severity of viral infections. Specifically, in SARS-CoV-2 infection, this correlation between HLAs and SARS-CoV-2 susceptibility and *Coronavirus* disease 2019 (COVID-19) severity has been highlighted, pointing out the relationship between particular alleles and severe infections [53]. Upregulated HLA molecules upon infections such as HLA-G membrane-bound, but mainly sHLA-G-, are potentially implicated in the suppression of immune system functions, including NK cells cytolysis, favoring immune tolerance to infections and thus virus subversion and replication during SARS-CoV-2 infections or COVID-19 disease [10,54]. Since the immune disability of NK cells has been related to interactions with its NKG2A/CD94 receptor that is upregulated in severe patients of COVID-19, the engagement of HLA-G to this receptor might be the cause of immune exhaustion observed in severe disease, which is highly dependent on key pathogen-induced cytokines and the binding affinity of different HLA-G alleles. In general, viral HLA-G upregulation can aggravate the morbidity of the virus and/or the mortality of the patient, such that HLA-G expression in these disorders may predict a worse outcome and increased susceptibility to cellular transformation [51].

The dynamics of peripheral immune cells, cytokines, and HLA-G and its receptor expressions have been described during critical COVID-19 pneumonia [55]. It has been suggested that the follow-up of sHLA-G could be used to identify the prognosis of COVID-19 among patients with high levels of sHLA-G [54,56]. It seems that HLA-G molecules control soluble Intercellular Adhesion Molecule-1 (sICAM-1) and sE-selectin expression through CD160 interaction and Fibroblast Growth Factor 2 (FGF2) induction and consequently lead to neutrophil adhesion and to the improvement of the disease outcomes [57]. The presence of HLA-G, as an important immune check point, might suggest the use of Immune-Checkpoint Inhibitors (ICIs) against this molecule in COVID-19 treatment [58].

HLA-G can interact with the inhibitory receptor Immunoglobulin-Like Transcript 2 (ILT2) in the blood and be secreted as a free soluble form (sHLA-G) or through extracellular vesicles [47]. Recently, new approaches revealed that the blockage of the HLA-G/ILT axis, could be insightful for the development of effective anti-tumor treatments strategies [59,60,61]. Since HLA-G exists in several polymorphs that affect both the protein expression levels and its peptide-binding cleft, targeting peptide binding cleft of the most common HLA-G polymorphs can also be suggested as a therapeutic strategy [31]. Moreover, it may be possible to modulate HLA-G transcription with microRNAs (miRNAs, miRs), such as hsa-miR-148a and miR-152, which bind to the 3′ untranslated region of the HLA-G gene (3’UTR), downregulating its mRNA levels [52,62]. Furthermore, mAs or RNA interfering strategies can be used to block HLA-G expression or HLA-G receptor, or the application of HLA-G-derived immunogenic peptide and anti-idiotype antibodies to activate immune cells can be fruitful approaches [15]. Since the option of blocking HLA-G using mAs appears unfeasible, mainly because they lack sufficient specificity, other options have recently been proposed. The core goal is directly blocking HLA-G binding with its receptors, and to this end, antibodies should be targeted for different key domains [14].

Therefore, based on the major immunomodulatory roles of HLA-G both in infectious and non-communicable diseases, we hypothesize a more-than-likely plausible capacity of key infections to upregulate HLA-G as therapeutic or immunomodulatory options, potentially allowing us to manage other infections.

**Table 1:** The correlation of HLA-G with infectious diseases caused by viruses, bacteria, and protozoan parasites.

Pathogens or Diseases	HLA-G	Observations	References
Hepatitis B virus (HBV)	HLA-G (14 bp ins/ins genotype)	Positive correlation with worse clinical manifestations	[63]
	sHLA-G	A significant correlation with the phase of HBV infection, clinical diagnosis, and disease persistence, and also progression toward hepatocellular carcinoma	[64]
Hepatitis C virus (HCV)	Higher levels of sHLA-G and interleukin-10 (IL-10)	Negative correlation with response to treatment	[65]
	HLA-G	A plausible function in the genesis of HCV liver fibrosis	[66]
Human immunodeficiency virus 1 (HIV-1)	HLA-G polymorphisms	HLA-G polymorphisms independently and synergistically induce susceptibility to heterosexual acquisition of HIV-1	[67]
Human Papillomavirus (HPV)	HLA-G polymorphism	Associations with the outcomes of oral HPV, affecting some characteristics of the women’s reproductive health, dual function (tumor progression and a good immunotherapeutic target) in cervical HPV	[68,69]
Herpesvirus (6A and 6B)	HLA-G	Inhibits in vitro angiogenesis through HLA-G	[70]
Cytomegalovirus (CMV)	HLA-G	Influences HLA-G expression in healthy individuals and probably contribute to viral immune evasion	[71]
	sHLA-G	As a promising biomarker of diagnosis of maternal CMV in maternal blood and amniotic fluid	[72]
Arbovirus	sHLA-G	A plausible biomarker to monitor neurological complications	[73]
*SARS-CoV-2*	HLA- G	The possible induction of profound immune suppression leading to the escape of virus from immune attack	[10]
*Helicobacter pylori*	HLA-G	Correlated with milder colonization and milder inflammation	[74]
*Pseudomonas aeruginosa*	sHLA-G	Decreasing levels during antibiotic therapy in patients with cystic fibrosis (negative correlation with inflammation)	[75]
	HLA-G	Inducing HLA-G expression in monocytes and T cells by *P. aeruginosa* (protecting from immune responses)	[76]
*Tropheryma whipplei*	HLA-G	Increased HLA-G levels in patients’ sera (promoting bacteria colonization)	[23]
Human African trypanosomiasis (HAT)	HLA-G 3′ UTR-2 and UTR-5 haplotypes	Association with increased susceptibility to HAT	[77]
	HLA-G 3′ UTR-4 haplotype	Association with a decreased risk of HAT	
	HLA-G 5′ URR-010102a/UTR-2 and 5′URR- 0103e/CR-G*01:03:01:02/UTR-5 haplotypes	Association with HAT disease progression	[78]
	HLA-G (rs1611139 T, rs17875389 A, rs9380142 G alleles)	Association with increased risk of infection	[79,80]
	HLA-G (rs1233330 A, rs1233330 G alleles)	Association with decreased risk of infection	[80,81]
American trypanosomiasis (Chagas disease)	Cell-surface HLA-G	Reduced HLA-G expression on cardiac muscle and colonic cells in patients with cardiac or digestive forms of Chagas, respectively	[82]
	HLA-G (+3003 T allele and +3003TT, +3187GG and +3196GC genotypes)	Association with an enhanced risk of symptomatic Chagas	
	HLA-G (+3003C allele and +3003CT and +3196CC genotypes)	Association with a decreased risk of symptomatic Chagas	
	HLA-G (+3027C and +3035C alleles and of +3027CC and +3035CC genotypes)	Association with the digestive form of Chagas	
Malaria	sHLA-G	Increased sHLA-G levels in cord blood and correlation with low weight at birth and clinical outcome (positive correlation with high risk of infection in infancy)	[83]
	HLA-G (+3187G allele and UTR1 haplotype)	Association with reduced level of parasite burden during *Plasmodium falciparum* asymptomatic infection	[84]
	HLA-G (UTR3 haplotype)	Association with enhanced level of parasite burden and increased severity of *P. falciparum* asymptomatic infection	
	HLA-G (+3010G and +3142C alleles)	Association with enhanced total IgG and IgG1 antibodies levels against *P. falciparum* glutamate-rich protein (GLURP)	[85]
	HLA-G (+3196G and UTR2 haplotype)	Association with a decreased IgG3 response against *P. falciparum* Merozoite Surface Protein 2 (MSP2)	
	sHLA-G	The correlation of increased sHLA-G levels in cord blood with low birth weight and an enhanced risk of malaria (*P. falciparum*) in the first year of life	[83,86]
	sHLA-G	The correlation of an increased mean sHLA-G levels during infancy with low birth weight and an enhanced risk of malaria (*P. falciparum*) during the two first years of life	[87]
Visceral leishmaniasis (VL)	sHLA-G	Increased levels of blood sHLA-G in *Leishmania*-infected patients	[88]
		Decreased levels of blood sHLA-G after anti-parasitic treatment of VL	[89]
*Toxoplasma* spp.	sHLA-G	Parasite increased the secretion of sHLA-G by trophoblast inducing apoptosis of decidual natural killer (dNK) cells (positive correlation with abnormal pregnancy)	[90]
		Increased sHLA-G levels in the amniotic fluid of pregnant women infected by *Toxoplasma* and in congenitally infected fetuses	[91]
		The increased secretion of sHLA-G by trophoblast due to *Toxoplasma* infection (inducing apoptosis of dNK cells)	[90]
	Cell-surface HLA-G	Increased HLA-G expression in the *Toxoplasma*-infected cells (decreasing HLA-G after treatment of infected cell by IL-10)	[92]

## 3. HLA-G Expression in Parasitic Diseases

It has been shown that high levels of sHLA-G are correlated with its involvement in the immune tolerance induced by hookworm’s infections during pregnancy [93]. In echinococcosis, the cyst activity may induce the release of sHLA-G in the blood, highlighting the presence of an immune-modulatory strategy, leading to the downregulation of the host inflammatory responses [94]. Increased levels of sHLA-G have been reported in patients presenting active phase of echinococcosis [39].

The expression of HLA-G gene and molecule has been also investigated in protozoan parasitic diseases including African trypanosomiasis, Chagas disease, malaria, toxoplasmosis, and leishmaniasis [39]. For instance, the enhanced expression level of sHLA-G was correlated with the increased predisposition to develop human African trypanosomiasis and promoted susceptibility to malaria, suggesting sHLA-G as a prognostic biomarker in such diseases [39,78]. The level of sHLA-G is also a crucial biomarker of the incidence of malaria during infancy and further confirm that mother sHLA-G levels could be considered a diagnostic marker of malaria susceptibility in children [95]. Moreover, sHLA-G also exerts a critical immune-modulatory function to decrease fetal loss due to toxoplasmosis infection; however, its overexpression could lead to a congenital transmission [91].

Furthermore, co-infections by several parasites are not uncommon. For example, those by *Schistosoma haematobium* and *Plasmodium falciparum* are frequent [96,97,98,99]. It has been shown that children with *S.* *haematobium* and *P. falciparum* coinfections exhibit lower expression levels of LILRB2, one of the receptors of HLA-G in B cells and neutrophils. However, its interaction with HLA-G led to complex sHLA-G derived immune regulatory pathways inducing effective neutralizing antimalarial humoral responses. Moreover, the impact of malaria on viral co-infections has been highlighted in patients with both diseases, suggesting a potential of malaria parasites to improve COVID-19 management due to their immune derived ability to influence virus clearance and pathology [100].

In addition, antibody responses to protective vaccine candidates for malaria are dependent on polymorphisms in the HLA-G gene. sHLA-G upregulation upon vaccination has shown important regulatory effects on the response to vaccination, with a blood-stage malaria vaccine candidate stimulating antigen presenting cells to secrete this molecule. Such a molecule seems to have a dual regulatory role, modulating anti-vaccine responses generating a decreased immunogenicity upon vaccination. These observations suggest that, depending on the levels of sHLA-G, the vaccine immunogenicity and parasite infection progression may change [86,101,102].

## 4. HLA-G as Target for Potential Immunotherapeutic Strategy

Recent data have elucidated the plausible antagonism between parasites and parasites, parasites and bacteria, and parasites and viruses (including *Plasmodium* against *Chikungunya virus*, *Heligmosomoides polygyrus* against *Respiratory Syncytial Virus*, and *Echinoparyphium* against *Ranavirus*) through different mechanisms including immunomodulation, vector modulation, interference competition, and exploitation competition. The immunomodulation strategy could happen in co-infections by the reduction in immunopathological changes caused by the Th1-type immune response [103]. Although some viruses such as *Herpesvirus B* and *SARS-CoV-2* stimulate the upregulation of HLA-G and HLA-E to facilitate immune escape [56,104], the induction (by different pathogens such as parasites) of a dramatic reduction in HLA-G expression levels in disorders or in pathogen-infected tissues might be significant.

In a recent study, it has been shown that there are common immune-dominant regions between *SARS-CoV-2* and *P. falciparum* that may explain the low COVID-19 incidence in the malaria-endemic areas [105]. Interestingly, a decreased membrane-bound HLA-G has been detected in the placenta of patients infected by *P. falciparum* and in the heart and colon of Chagas disease patients [39]. Although the parasite may have a possible dual role regulating HLA-G, it seems that *P. falciparum* infection is able to downregulate HLA-G production (42% vs. 90% in controls) in extravillous cytotrophoblast cells in correlation with the increased presence of NK cells [106]. The increased number of NK cells is probably related to the direct or indirect action of some cytokines, especially the pro-inflammatory IL-10 produced by a large variety of cells such as macrophages, which also induce NK-cell proliferation [107]. Numerous pathogens, including malaria parasites, induce high levels of proinflammatory cytokines by the upregulation of co-inhibitory receptors [108]. These inhibitory receptors are present in unconventional T cells, pointing out the high potential of these cells in infectious diseases [109].

Both IL-10 and *Transforming Growth Factor Beta* (TGF-β) act as important anti-inflammatory immunomodulators helping to control malaria. Furthermore, at the early stages of SARS-CoV-2 infection, TGF-β upregulation and downstream signals have antiviral implications, as demonstrated with artemisin therapy. Moreover, there are data supporting the correlating levels of total sHLA-G in plasma with elevated TGF-β levels in systemic sclerosis, TGF-β also being one major factor upregulating HLA-G expression in cancers, and subsequently influencing immune cells mainly Treg cells and, on the other hand, participating in cancer immune escape and immune checkpoint resistance [110,111]. Thus, we speculate that the key immunomodulatory role of the cytokines TGF-β and IL-10 and the essential regulation of T cells including Treg cells by TGF-β might initially be linked molecularly at least during the early phase of parasite infections with HLA-G levels. This could be most probably through indirect regulatory interactions with the pathway (involving HLA-G) finally affecting different immune cells [112]. Publications with data of high levels of HLA-G, IL-10, and TGF-β bearing therapeutic exosomes improving disease symptoms together with peripheral blood cells unresponsiveness might support this molecular pathway [113]. In this sense, demonstrating the implication of HLA-G levels in TGF-β-derived signaling could serve as a novel and important therapeutic strategy [114,115]. From this angle, *P. falciparum* or special antigens related to this parasite might be involved in the HLA-G reduction in extravillous cytotrophoblast cells. Although the mechanisms leading to such conditions remain unknown, experimental studies in this regard could further highlight this issue and might suggest the use of parasites including *P. falciparum* in the modulation (reduction) of HLA-G expression in other tissues, such as tissues infected by *SARS-CoV-2*. Since impaired NK cell counts and the cytolytic activity of these cells are important characteristics of severe COVID-19 [116,117], the increased levels of NK cells induced by *P. falciparum* might be considered a potential mechanism and strategy related to immune-modulatory-based therapies against COVID-19.

## 5. Conclusions

Taking into account that, in parasite endemic areas of COVID-19 cases, the severity of the disease and numbers of deaths decreased mainly due to patient coinfections with parasites, it seems that the effect of some pathogens such as parasites restricting HLA-G expression can be further considered in future studies [118,119]. Unlike other viral or bacterial coinfections that negatively impact respiratory viral diseases such as COVID-19, some parasitic infections could modulate disease severity and the clinical outcome of other infectious diseases such as COVID-19 via intricate molecular networks involving HLA-G, IL-10/TGF-β, and Treg cells [103,118,120]. Finally, as master immune modulators, their levels may be critical for the development of proper immune responses to vaccination, thus serving as potential targeting molecules to achieve desirable outcomes. These data highlight the importance of HLA-G molecule immune modulation in the context of previous infections, revealing the promising outlook for future research on this molecule and its potential ability to understand pathogen interactions and the molecular mechanism underlying the specific criteria of suitable helminths for therapy and to manage COVID-19 disease progression and pathogenesis. This would be assessed adapting appropriate experimental model systems of coinfections which might also help to reinforce the idea of parasites more than their products, keeping proper immune function and the need of controlled exposures that may render us less susceptible to pandemics or chronic inflammatory diseases [121].

## Data Availability

Not applicable.
